# Crystal structure of the bifunctional enzyme phosphoribosyl isomerase A from *Mycobacterium tuberculosis* with bound light-controllable inhibitor

**DOI:** 10.1107/S2053230X26009684

**Published:** 2026-10-12

**Authors:** Chitra Rajendran, Reinhard Sterner

**Affiliations:** a Institute of Biophysics and Physical Biochemistry, Regensburg Center for Biochemistry University of Regensburg 93040Regensburg Germany; National Centre for Biological Sciences-TIFR, India

**Keywords:** phosphoribosyl isomerases, enzyme inhibition, light-controllable enzymes, molecular switches, PriA

## Abstract

The enzyme phosphoribosyl isomerase A from *Mycobacterium tuberculosis* (mtPriA) catalyzes two related reactions of bi- and monophosphorylated substrates within the histidine and tryptophan biosynthetic pathways. We have determined the crystal structure of mtPriA with a bound light-controllable 1,2-dithienylethene inhibitor (DTE LC6) at a resolution of 1.95 Å. DTE LC6 contains two terminal phosphate groups, and we found one inhibitor molecule (DTE LC6-1) simultaneously bound to the two known functional phosphate-binding sites of mtPriA. Unexpectedly, another inhibitor molecule (DTE LC6-2) was shown to bind to a hitherto unidentified third phosphate-binding site and thus bridges two crystallographic symmetry-related mtPriA proteins. Our study explains the previously reported inhibitory effect of DTE LC6 on the catalytic activity of mtPriA and raises the question of the significance of the third phosphate-binding site in mtPriA.

## Abstract 2

We have determined the crystal structure of an isomerase catalyzing two steps in histidine and tryptophan biosynthesis with a bound light-switchable competitive inhibitor. The work explains the mode of action of the inhibitor and describes the discovery of a hitherto unknown third phosphate-binding site of the enzyme.

## 1. Introduction

**Figure 1:**
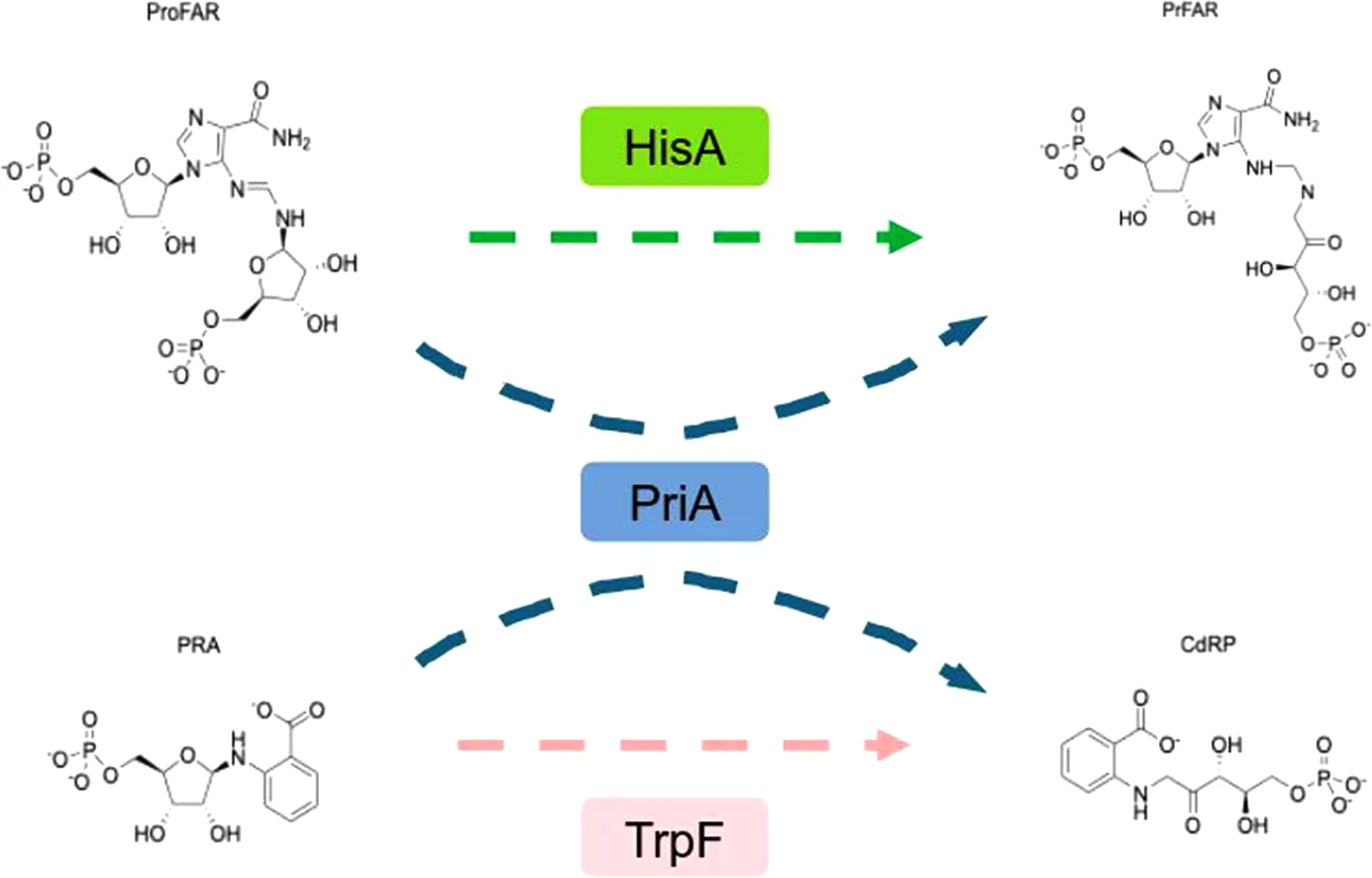
Reaction schemes for HisA, TrpF and PriA.

In most bacteria, the histidine (His) and tryptophan (Trp) biosynthesis pathways involve two similar Amadori rearrangements in which an aminoaldose is converted into the corresponding aminoketose. Whereas the enzyme HisA isomerizes ProFAR (*N*′-[(5′-phosphoribosyl)formimino]-5-aminoimidazole-4-carboxamideribonucleotide) into PRFAR (*N*′-[(5′-phosphoribulosyl)formimino]-5-aminoimidazole-4-carboxamide ribonucleotide), the enzyme TrpF isomerizes phosphoribosyl anthranilate (PRA) into 1-(*o*-carboxyphenyl-amino)-1-deoxyribulose 5-phosphate (CdRP). Remarkably, in most known actinobacteria a gene encoding TrpF is missing and a bifunctional enzyme called PriA catalyzes both the TrpF and HisA reactions (Barona-Gómez & Hodgson, 2003 ▸; Fig. 1 ▸). As humans can synthesize neither histidine nor tryptophan, mtPriA is considered to be a potential target for antituberculosis drugs (Mdluli & Spigelman, 2006 ▸).

**Figure 2:**
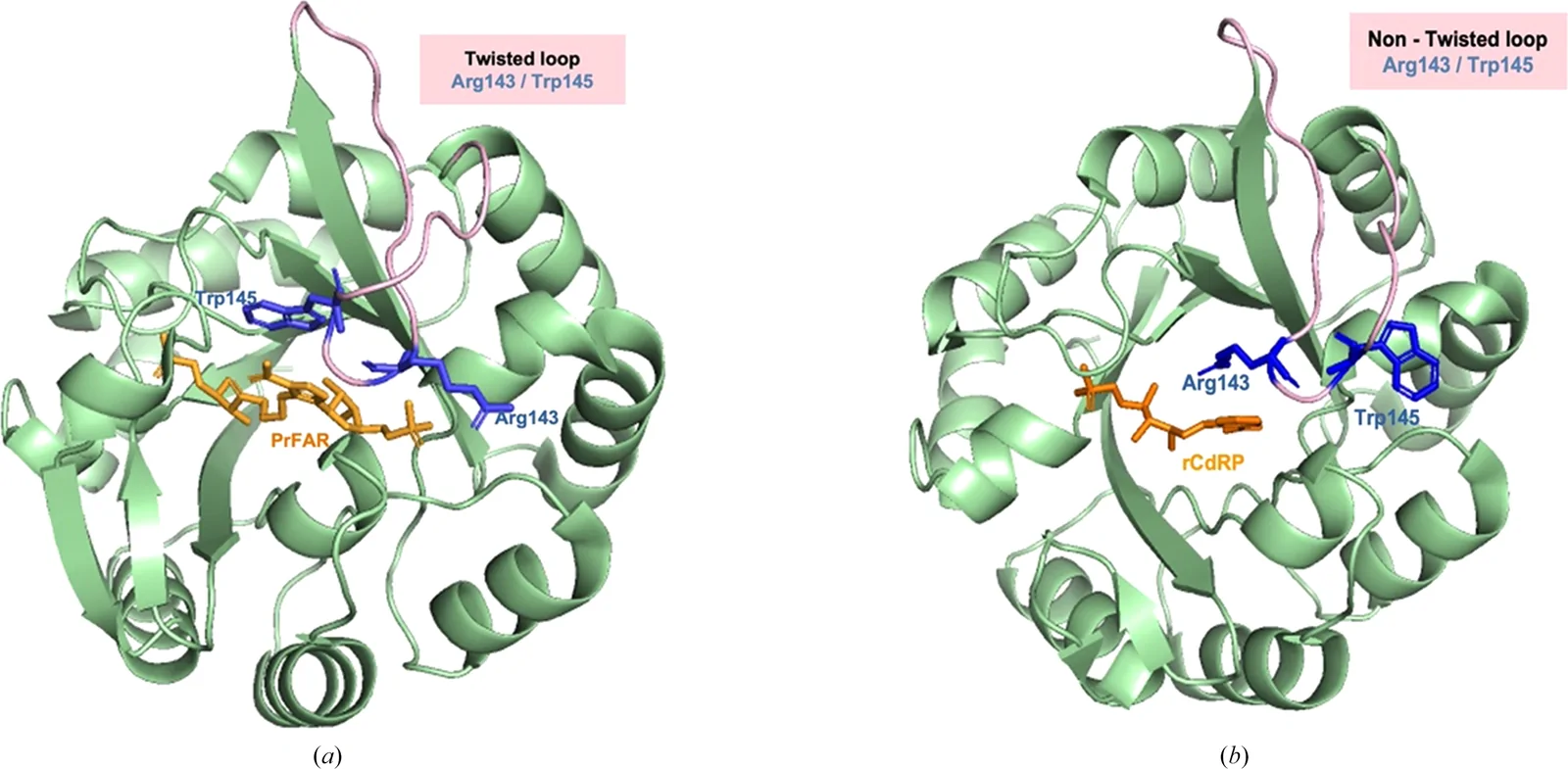
(*a*) Cartoon representation of mtPriA (pale green) with PRFAR (orange) bound to the N- and C-terminal phosphate-binding sites (PDB entry 3zs4). Loop 5 is highlighted in pink, and residues Arg143/Trp145 involved in twisting loop 5 are shown in blue. (*b*) Cartoon representation of mtPriA (pale green) with rCdRP (orange) bound to the C-terminal binding site (PDB entry 2y85). Loop 5 is highlighted in pink and residues Arg143/Trp145 are shown in blue.

TrpF, HisA and PriA belong to the class of (βα)_8_-barrel proteins, which is a frequently encountered and highly versatile enzyme fold (Sterner & Höcker, 2005 ▸). Moreover, like HisA and HisF, which catalyzes the following step in histidine biosynthesis, the structurally characterized PriA from *Mycobacterium tuberculosis* (mtPriA) exhibits a twofold symmetry (Due *et al.*, 2011 ▸), indicating evolution from a (βα)_4_ half-barrel precursor (List *et al.*, 2011 ▸). Previously, structures of mtPriA were determined with the bound HisA product PRFAR and TrpF product analog reduced CdRP (rCdRP), respectively. The results showed that mtPriA contains an N-terminal and a C-terminal phosphate-binding site that are involved in recruiting the two phosphate groups of PRFAR (Fig. 2 ▸*a*). The single phosphate group of rCdRP is recruited by the C-terminal phosphate-binding site of mtPriA (Fig. 2 ▸*b*).

The artificial control of the activity of macromolecules by light is an advancing area in molecular biology. Light regulation of biomolecules can be achieved by several strategies such as reprogramming naturally occurring photoreceptors, functionalizing key positions in the molecule with photolabile protecting groups, directing cellular or protein function by photoswitches and modification of the active sites of enzymes (Kneuttinger, 2022 ▸).

**Figure 3:**
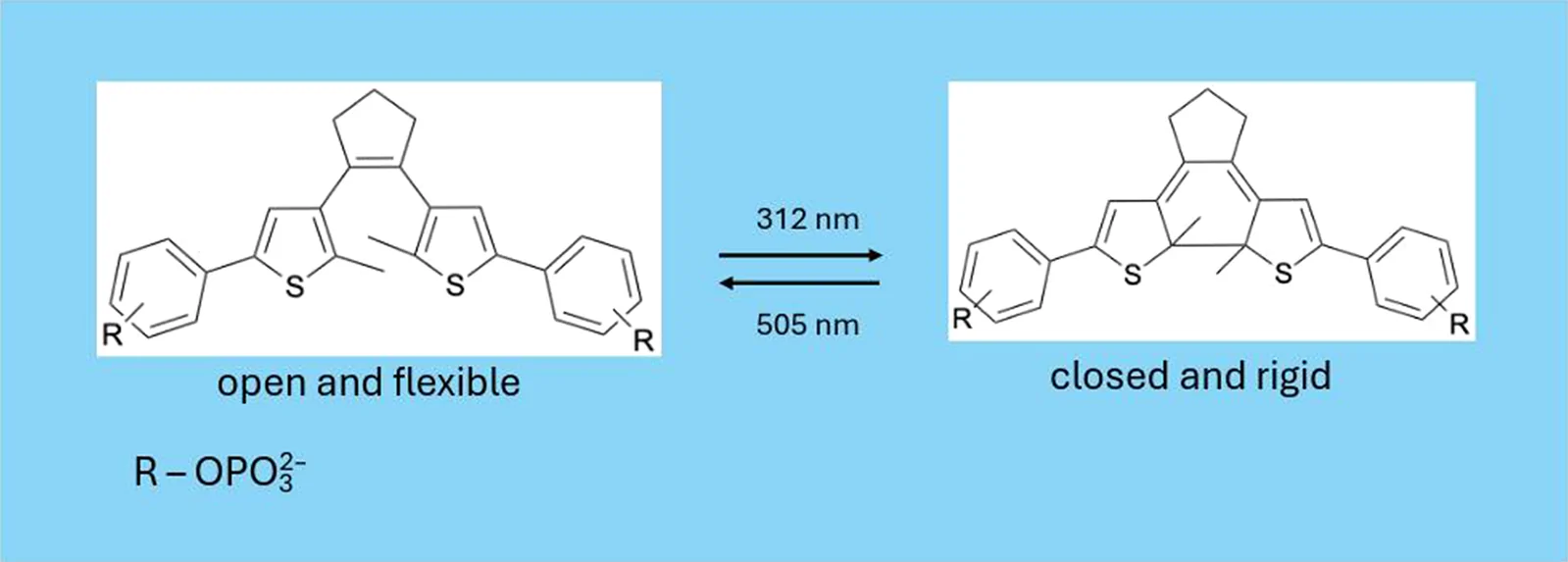
DTE ligands with terminal phosphate groups. The open and flexible forms are reversibly converted into the closed and rigid forms, respectively, by light at the indicated wavelengths.

Active-site modifications can be achieved by the covalent attachment of light-responsive elements such as caged un­natural amino acids (Luo *et al.*, 2017 ▸). Alternatively, non­covalently bound molecules such as 1,2-dithienylethene (DTE) ligands can be used to govern enzymatic activity (Szymański *et al.*, 2013 ▸; Feringa, 2007 ▸). DTEs, which are a subgroup of diarylethene ligands and show a twofold symmetry, are thermally stable up to 80°C and feature reversible photoconversions of >90% (Vomasta *et al.*, 2008 ▸; Irie, 2000 ▸). They are composed of two thiophene moieties linked by an ethene unit that is incorporated into a cyclopentene ring to prevent *Z*-to-*E* isomerization. Upon irradiation with UV light DTE undergoes a cyclization reaction towards a ring-closed isomer, whereas visible light populates the ring-open isomer (Vomasta *et al.*, 2008 ▸; Fig. 3 ▸).

Taking advantage of the twofold symmetries of both mtPriA and DTEs, we have previously designed competitive DTE inhibitors with different phosphate and phosphonate anchors that were bound by the two phosphate-binding sites of mtPriA (Reisinger *et al.*, 2014 ▸). The DTE molecule with the highest affinity was called compound 6 (DTE LC6). It bound to the active site of mtPriA with *K*_i_ = 0.55 µ*M* in its flexible open configuration and *K*_i_ = 4.4 µ*M* in its rigid closed configuration. These different affinities allowed us to control the conversion of ProFAR into PRFAR by mtPriA with light. When the reaction was started with DTE LC6 in its strongly inhibiting open form, the catalytic rate could be enhanced by about threefold upon switching to the lower affinity ring-closed isomer. In the current work, we have used X-ray crystallo­graphy to analyze the mode of binding of DTE LC6 to mtPriA.

## 2. Materials and methods

### 2.1. Production and crystallization of mtPriA and DTE LC6

The cloning, heterologous expression in *Escherichia coli* and purification of mtPriA, as well as the production of the inhibitor DTE LC6, were performed as described in Reisinger *et al.* (2014 ▸).

Initial crystallization trials were carried out using commercially available PEG screens. The hanging-drop vapor-diffusion method was performed in 96-well plates (Greiner) at 291 K. Crystallization was carried out in dark-room conditions using red LED safe lights, and the plates were wrapped in multiple layers of aluminium foil. The DTE LC6 ligand was co-crystallized with the protein at a twofold molar excess. Drops contained a 1 µl:1 µl ratio of the respective reservoir buffer and protein plus ligand, respectively. In each well, equilibration was performed against 100 µl reservoir buffer. Crystals were obtained with 0.15 *M* lithium sulfate monohydrate, 0.1 *M* citric acid pH 3.5, 20%(*w*/*v*) PEG 6000. After flash-cooling in liquid nitrogen, data were collected from single crystals on the synchrotron beamlines PXI and PXIII at the Swiss Light Source (SLS) at 100 K*.*

### 2.2. Data collection, structure solution and refinement

Data collection was performed on beamlines PXIII and PXI at SLS at cryogenic temperature. Data processing was performed using *XDS* (Kabsch, 1993 ▸, 2010*a* ▸,*b* ▸) and data-quality assessment was performed using *phenix.xtriage*. Structure determination was performed by molecular replacement with *Phaser* (McCoy *et al.*, 2007 ▸) using PDB entry 3zs4 (A. V. Due, J. Kuper, A. Geerlof & M. Wilmanns, unpublished work) as a template or search model. The model was further improved in several rounds of refinement using automated restrained refinement with *Phenix* (Adams *et al.*, 2002 ▸, 2010 ▸, 2011 ▸; Liebschner *et al.*, 2019 ▸) and interactive modeling with *Coot* (Emsley & Cowtan, 2004 ▸; Emsley *et al.*, 2010 ▸). The refinement statistics are listed in Table 1 ▸. The final model was analyzed using *MolProbity* (Davis *et al.*, 2007 ▸; Williams *et al.*, 2018 ▸) and has been deposited in the Protein Data Bank (PDB entry 30hl). Polder omit maps (Liebschner *et al.*, 2017 ▸) were created by *phenix.polder*.

## 3. Results and discussion

### 3.1. Structure of mtPriA with bound light-controllable inhibitor DTE LC6

**Figure 4:**
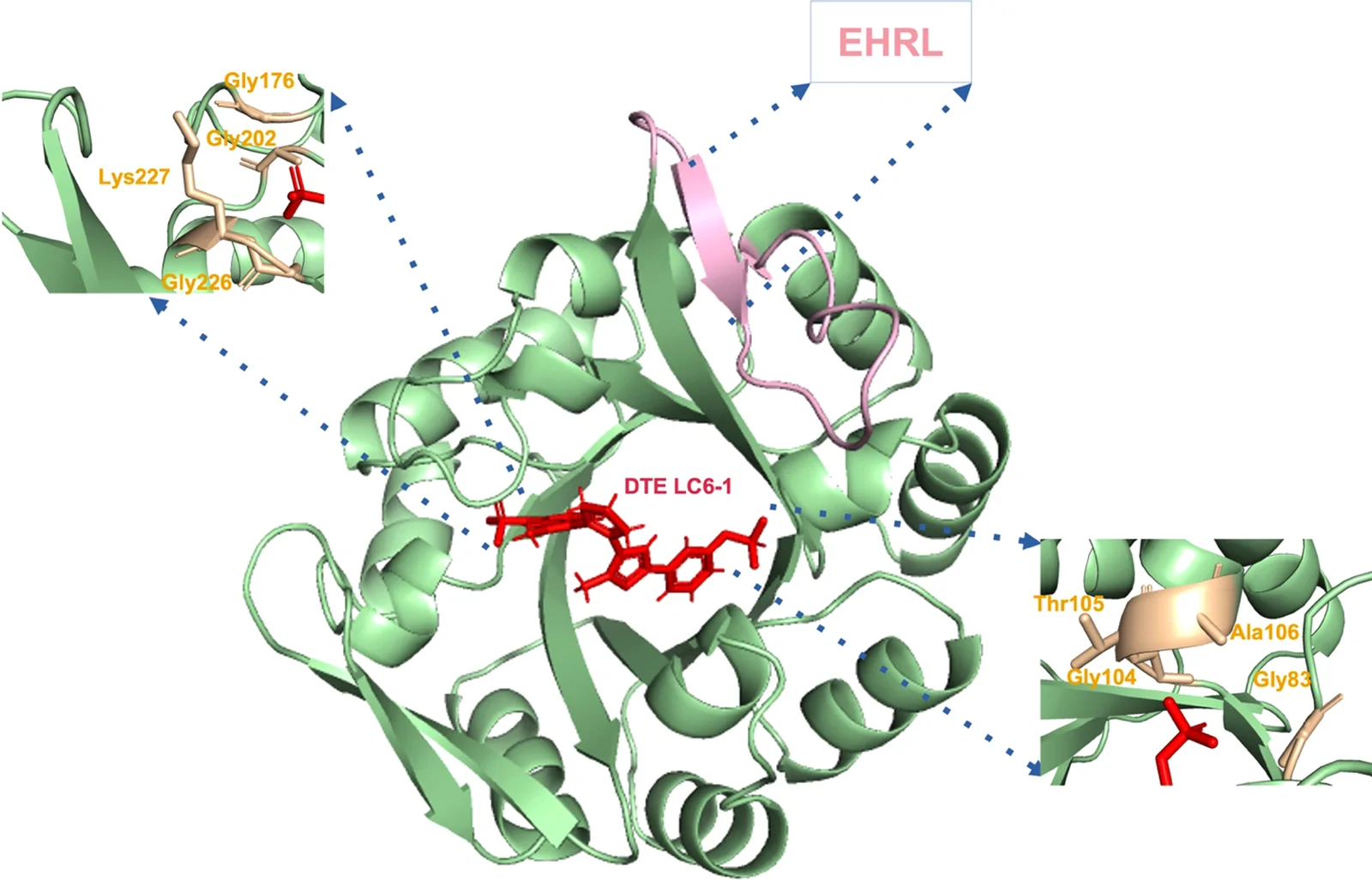
Cartoon representation of mtPriA (pale green) with bound inhibitor DTE LC6-1 (red). Loop 5 with residues EHRL forming an additional short β-strand is highlighted in pink. In the insets, the residues in wheat show the N-terminal (Gly83, Gly104, Thr105, Ala106) and C-terminal (Gly176, Gly202, Gly226, Lys227) residues involved in binding of the terminal phosphates of DTE LC6-1 (see Table 2 ▸ and Supplementary Fig. S2).

We determined the structure of mtPriA with bound light-controllable inhibitor DTE LC6 in its open form at a resolution of 1.95 Å (Fig. 4 ▸). The refinement statistics are shown in Table 1 ▸.

The structure was compared with the structures of mtPriA with bound PRFAR and of mtPriA with bound rCdRP, with a focus on the residues involved in binding the phosphate group(s) of the ligands (Table 2 ▸). The two terminal phosphate groups of DTE LC6 are complexed via hydrogen bonds by an N-terminal (Ser81, Gly83, Thr105, Ala106) and a C-terminal (Gly202, Gly176, Gly226, Lys227) phosphate-binding site, similarly to as observed in the PRFAR-bound structure (Fig. 2 ▸*a*). Interestingly, when compared with the two published liganded structures of mtPriA, loop 5 in mtPriA–DTE LC6 contains an extra β-strand formed by residues 137–140 (EHRL). Moreover, whereas in mtPriA–PRFAR and mtPriA–rCdRP loop 5 folds over the active site, representing a closed form (Figs. 2 ▸*a* and 2 ▸*b*), in mtPriA–DTE LC6 the loop is slightly disoriented, representing an open form (Fig. 4 ▸). Furthermore, in mtPriA with bound PRFAR, loop 5 twists into a knot-like conformation, which causes a position exchange of residues Arg143 and Trp145, called the ‘Arg143/Trp145 switch’ (Due *et al.*, 2011 ▸; Fig. 2 ▸*a*). In contrast, there is no knot and therefore also no Arg143/Trp145 switch in mtPriA with bound rCdRP (Fig. 2 ▸*b*) or bound inhibitor DTE LC6 (Fig. 4 ▸). The differences between loop 5 in mtPriA–PRFAR and mtPriA–DTE LC6 are highlighted in a superposition of the structures shown in Supplementary Fig. S1.

**Figure 5:**
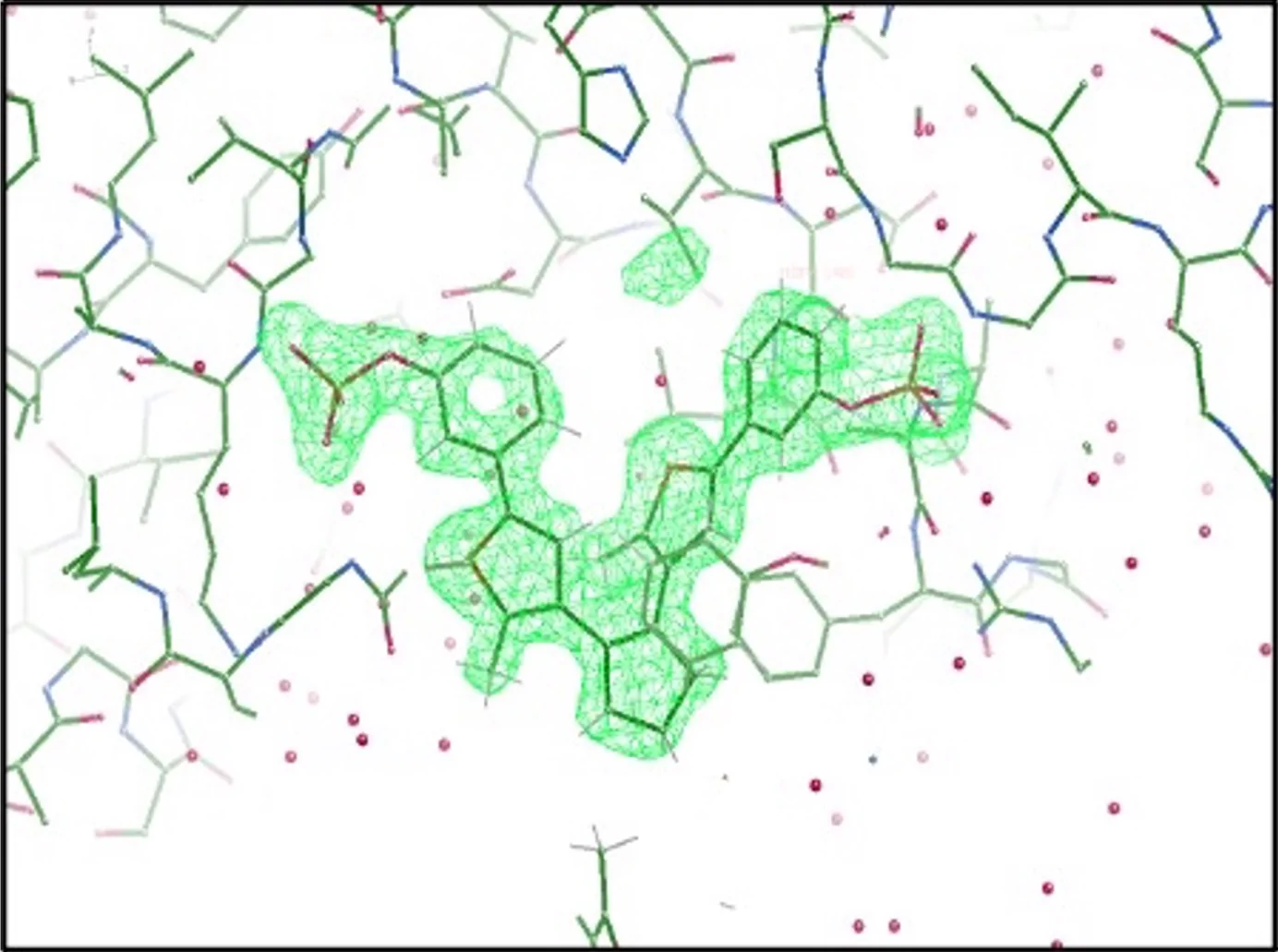
Polder omit difference electron-density map of the DTE LC6-1 binding site. The *mF*_o_ − *DF*_c_ polder omit electron-density map calculated using *phenix.polder* is shown as a green mesh contoured at 3σ.

A polder omit electron-density map of the bound ligand DTE LC6 is shown in Fig. 5 ▸.

### 3.2. A second inhibitor molecule connects two mtPriA proteins

**Figure 6:**
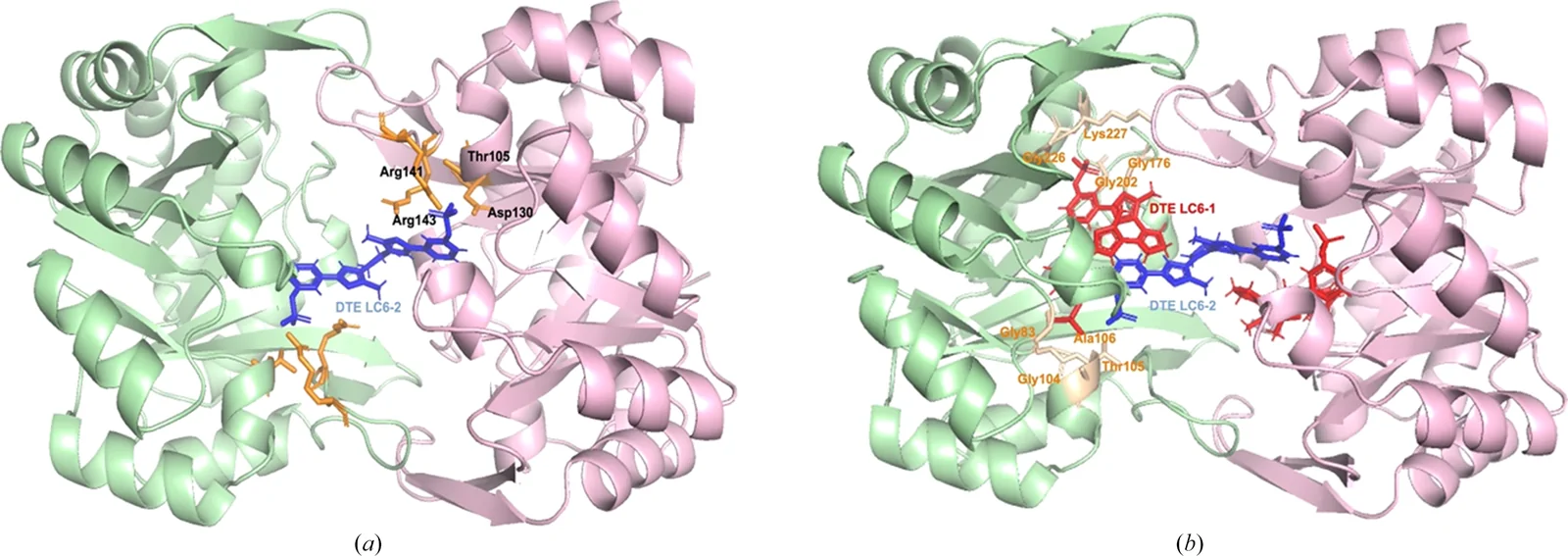
(*a*) Cartoon representation of two crystallographic symmetry-related mtPriA molecules (pale green and pink) connected by bound inhibitor DTE LC6-2 (blue). The additional phosphate-binding site residues (Thr105, Asp130, Arg141, Arg143) in both mtPriA molecules are shown in wheat. (*b*) Cartoon representation of two crystallographic symmetry-related mtPriA molecules (pale green and pink) with bound inhibitors DTE LC6-1 (red) and DTE LC6-2 (blue). The N-terminal (Gly83, Gly104, Thr105, Ala106) and C-terminal (Gly176, Gly202, Gly226, Lys227) phosphate-binding site residues of mtPriA are shown in wheat (see Table 2 ▸ and Supplementary Figs. S2 and S3).

Intriguingly, the open conformation of loop 5 in mtPriA–DTE LC6 not only enables the formation of a new small β-sheet but also exposes a new (third) phosphate-binding site including residues Thr105, Asp130, Arg141 and Arg143 (Fig. 6 ▸*a*).

Since the crystallographic symmetry-related mtPriA protein contains the same third phosphate-binding site, a second bound DTE LC6 inhibitor (DTE LC6-2) connects these two mtPriA molecules (Fig. 6 ▸*b*).

The residues of mtPriA engaged in the binding of the two DTE LC6 molecules (DTE LC6-1 and DTE LC6-2) are listed in Table 2 ▸.

We cannot definitely exclude that the third phosphate-binding site is a crystallographic artifact. However, the finding that DTE LC6-2 binds to the same site in both symmetry-related mtPriA molecules and via a similar number of well defined hydrogen bonds as does DTE LC6-1 (see Supplementary Figs. S2 and S3) would seem to indicate a specific binding. The function of the third phosphate-binding site can currently only be speculated on. It could, for example, help to recruit the TrpF substrate PRA and/or the HisA substrate ProFAR, or could allow the products CdRP/PRFAR to escape from the active site.

### 3.3. Conformational flexibility of light-controllable inhibitor

The DTE LC6 inhibitors have two terminal phosphates (Fig. 6 ▸*a*), and the calculated P–P distance depicting the open state of the inhibitor is shown in Table 3 ▸. Based on this, we checked the P–P distance of the two bound inhibitors, DTE LC6-1 and DTE LC6-2, in the structure of mtPriA. The P–P distance for DTE LC6-1 is 14.7 Å and that for DTE LC6-2 is 16.2 Å. The difference in the P–P distance of the bound inhibitor testifies to its conformational flexibility. The inhibitor DTE LC6-1 is fixed between the two conserved phosphate-binding sites in the core of mtPriA and is therefore quite rigid. In contrast, the inhibitor DTE LC6-2 is bound between the two flexible loops 5 of two molecules of mtPriA. This gives it more flexibility and increases the P–P distance of DTE LC6-2 compared with DTE LC6-1.

## Supplementary Material

PDB reference: inhibitor-bound PriA, 30hl

Supplementary Figures. DOI: 10.1107/S2053230X26009684/vo5026sup1.pdf

## Figures and Tables

**Table 1:** Data-collection and refinement statistics for mtPriA with bound DTE LC6-1 and DTE LC6-2 Values in parentheses are for the highest resolution shell.

Wavelength (Å)	1.00
Resolution range (Å)	44–1.949 (1.99–1.95)
Space group	*P*2_1_22_1_
*a*, *b*, *c* (Å)	47.23, 110.56, 121.16
α, β, γ (°)	90, 90, 90
Unique reflections	46723 (2591)
Completeness (%)	98.91 (94.39)
Wilson *B* factor (Å^2^)	12.57
Reflections used in refinement	46723 (2591)
Reflections used for *R*_free_	2337 (129)
*R*_work_	0.1787 (0.2250)
*R*_free_	0.2218 (0.2554)
No. of non-H atoms	
Total	4460
Macromolecules	3582
Ligands	117
Solvent	761
Protein residues	486
R.m.s.d., bonds (Å)	0.006
R.m.s.d., angles (°)	0.74
Ramachandran favored (%)	97.93
Ramachandran allowed (%)	2.07
Ramachandran outliers (%)	0.00
Rotamer outliers (%)	0.00
Clashscore	2.12
Average *B* factor (Å^2^)	
Overall	14.94
Macromolecules	13.02
Ligands	10.40
Solvent	24.66

**Table 2:** mtPriA residues involved in the binding of the phosphate moieties of the two DTE LC6 molecules Hydrogen-bond interactions as defined by *PyMOL* (Schrödinger) between side-chain or main-chain atoms of the listed residues and O atoms of the ligand phosphate are shown in Supplementary Figs. S2 and S3.

Ligand	N-terminal phosphate-binding site	C-terminal phosphate-binding site	Additional phosphate-binding site in crystallographic symmetry-related mtPriA molecules
rCdRP	—	Gly202, Gly176, Gly226, Lys227	—
PRFAR	Gly83, Arg85, Gly104, Thr105	Gly202, Gly176, Gly226, Lys227	—
DTE LC6-1	Gly83, Gly104, Ala106, Thr105	Gly202, Gly176, Gly226, Lys227	—
DTE LC6-2	—	—	Thr105, Asp130, Arg141, Arg143

**Table 3:** Phosphate–phosphate distances of ligands in different mtPriA structures

PRFAR	16.9 Å
DTE LC6-1	14.7 Å
DTE LC6-2	16.2 Å
