# Supplementary material for: Crystal structure of the bifunctional enzyme phosphoribosyl isomerase A from Mycobacterium tuberculosis with bound light-controllable inhibitor
Source: Acta Crystallogr F Struct Biol Commun. 2026 Oct 12;82(Pt 11):385–90. doi: 10.1107/S2053230X26009684 (PMC13717835; doi:10.1107/S2053230X26009684)
Supplement: Supplementary file 1 [file f-82-00385-sup1.pdf]

STRUCTURAL BIOLOGY
COMMUNICATIONS

**Volume 82 (2026)**

**Supporting information for article:**

# Crystal structure of the bifunctional enzyme phosphoribosyl isomerase A from *Mycobacterium tuberculosis* with bound light-controllable inhibitor

**Chitra Rajendran and Reinhard Sterner**

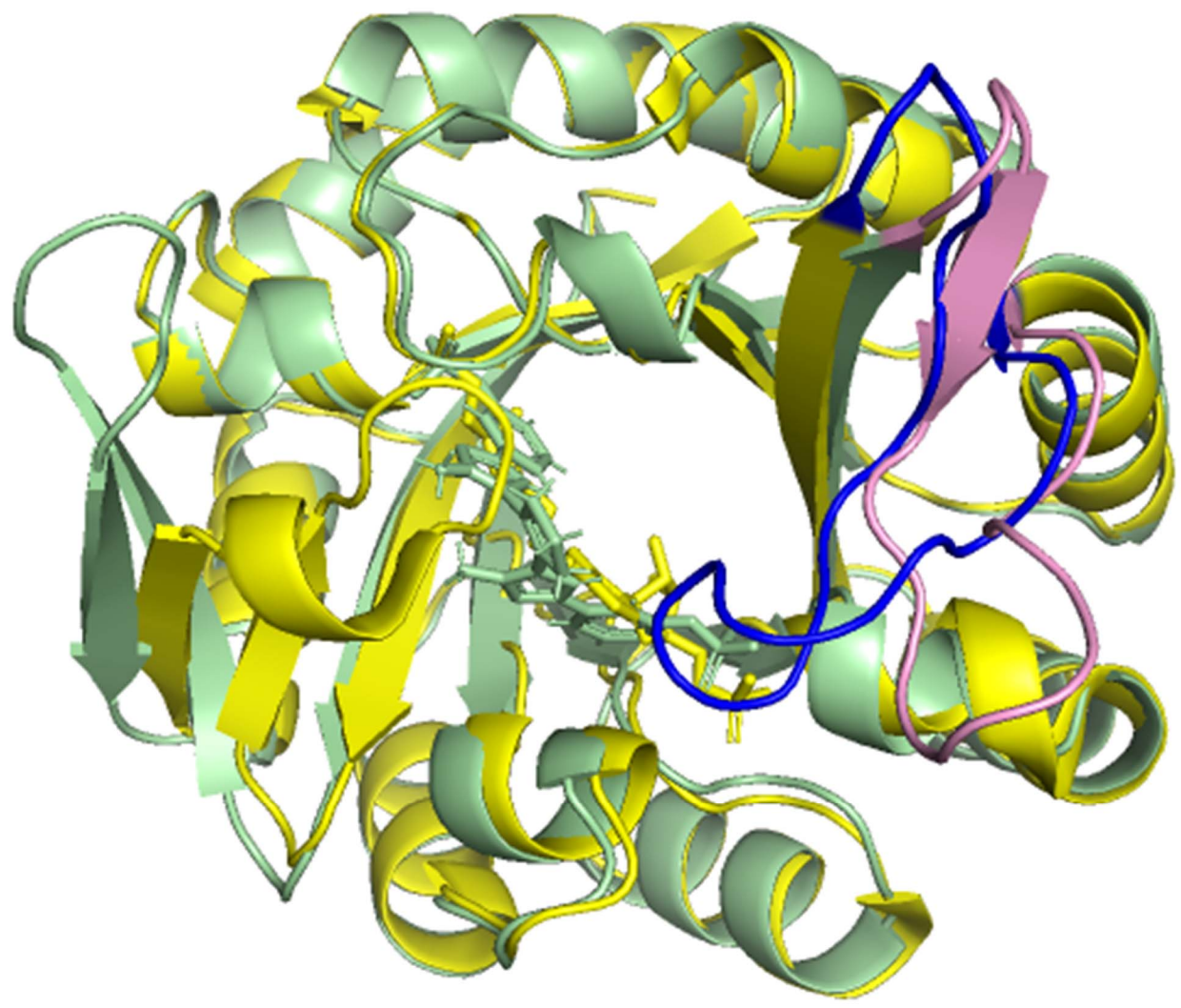

**Figure S1** Cartoon representation of a superposition of mtPriA structures with bound inhibitor DTE LC6-1 (pale green) and bound product PRFAR (yellow). Loop 5 containing an additional short β-strand in mtPriA-bound DTE LC 6 is highlighted in pink and loop5 with bound PRFAR is shown in blue.

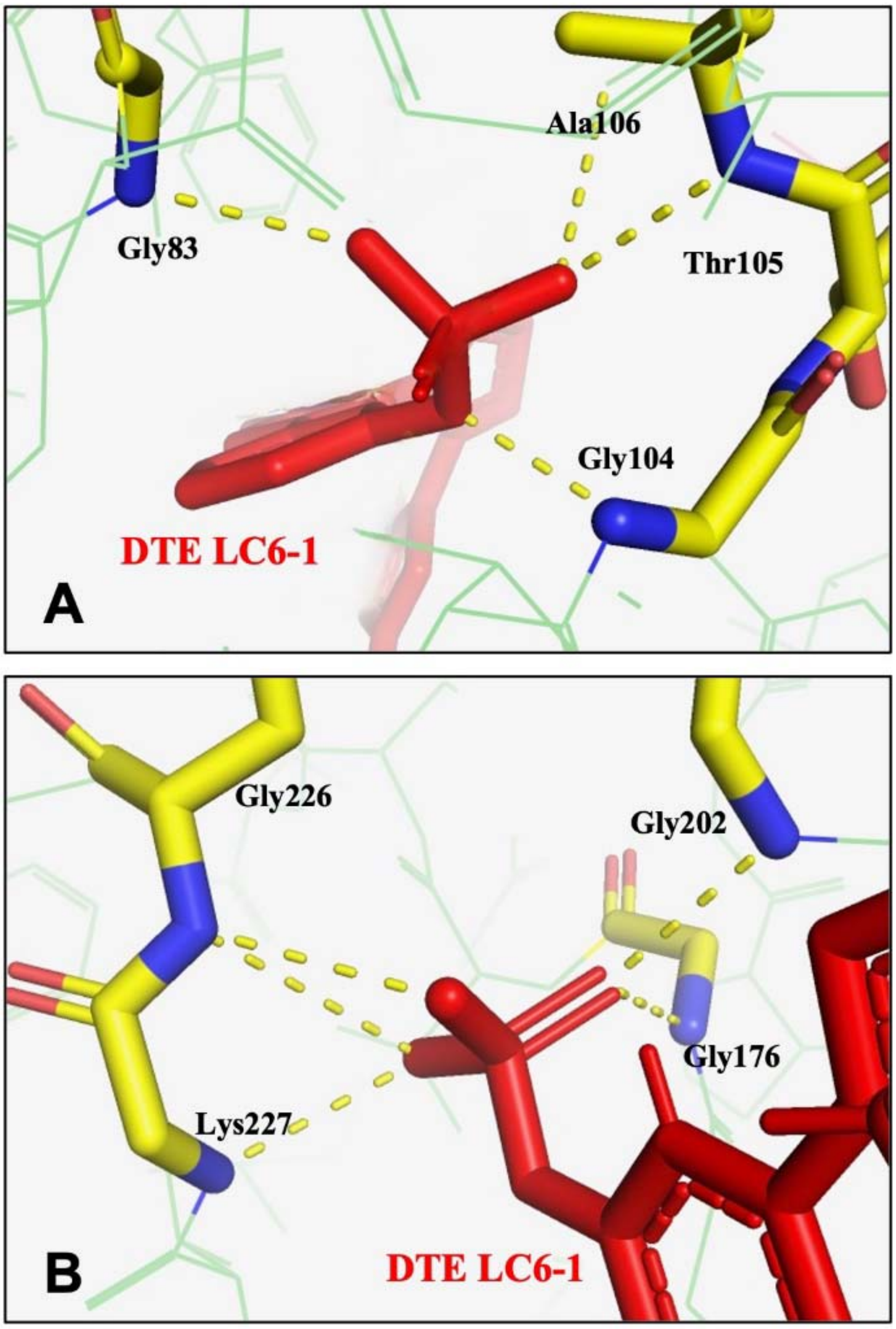


**Figure S2** Cartoon representation of interactions of ligand DTE LC6-1 with the N-and C-terminal phosphate-binding sites in mtPriA. (**A**) N-terminal phosphate-binding residues Gly83, Gly104, Thr105, Ala106 and (**B**) C-terminal phosphate-binding residues Gly176, Gly202, Gly226, Lys227 of mtPriA are colored by element. DTE LC6-1 is represented in red. H-bond interactions between phosphate groups of the ligand and the residues are shown in yellow.

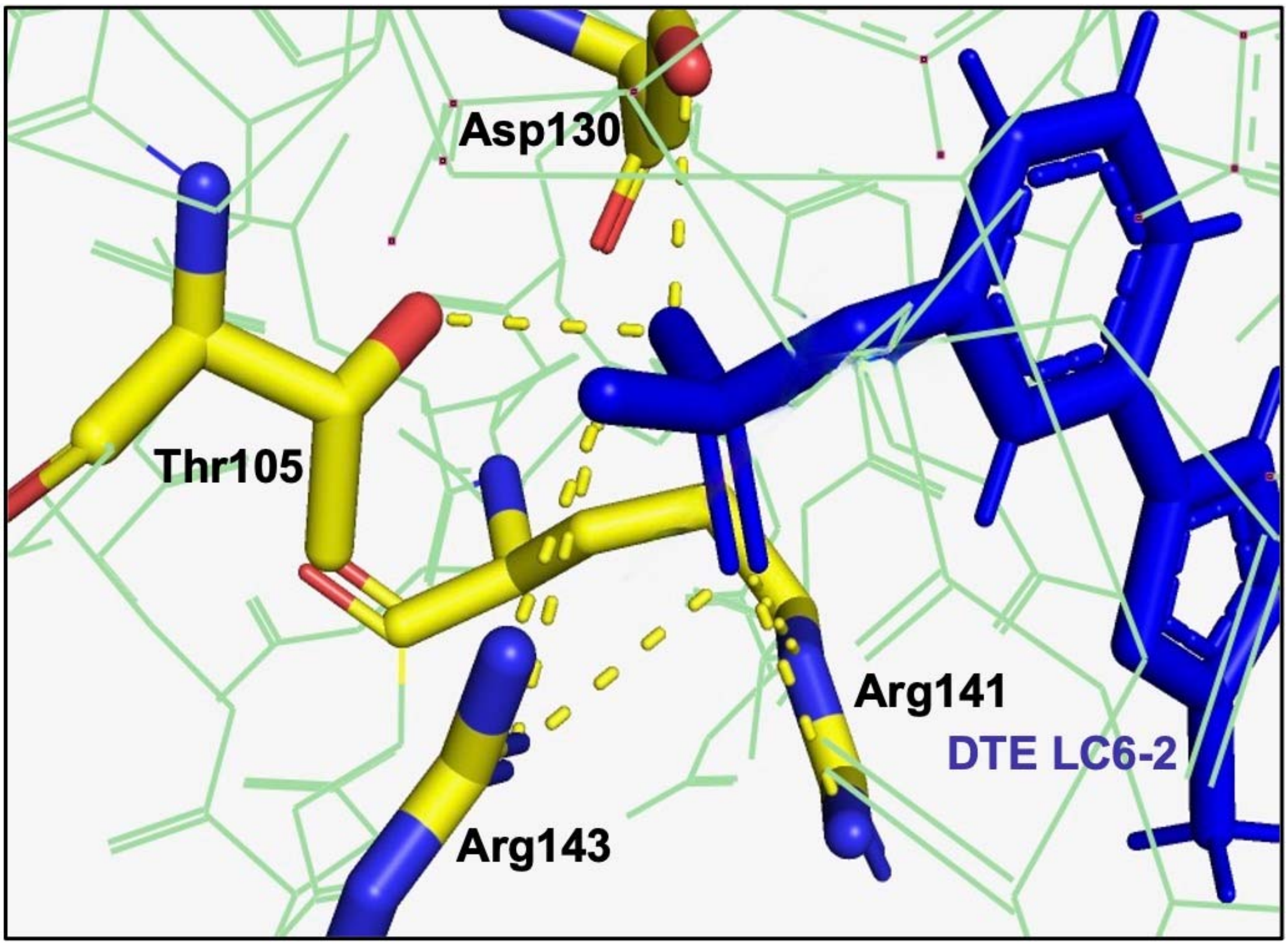


**Figure S3** Cartoon representation of interactions of ligand DTE LC6-2 with the third phosphate-binding site residues Thr105, Asp130, Arg141, Arg143 in mtPriA colored by element. DTE LC6-2 is represented in blue. H-bond interactions between phosphate groups of the ligand and the residues are shown in yellow.
